# Borderline Personality Disorder in a “Life History Theory” Perspective: Evidence for a Fast “Pace-of-Life-Syndrome”

**DOI:** 10.3389/fpsyg.2021.715153

**Published:** 2021-07-26

**Authors:** Benjamin Otto, Lisa Kokkelink, Martin Brüne

**Affiliations:** LWL University Hospital Bochum, Department of Psychiatry, Psychotherapy and Preventive Medicine, Division of Social Neuropsychiatry and Evolutionary Medicine, Ruhr-University Bochum, Bochum, Germany

**Keywords:** borderline personality disorder, life history theory, pace of life, early adversity, chronic stress, aggressiveness, personality traits, allostatic load

## Abstract

“Borderline Personality Disorder” (BPD) is associated with heightened risk for cardiovascular disease and other stress-associated somatic consequences, which is poorly understood in terms of causal mechanisms, such as childhood trauma. Here, we tested the hypothesis suggesting that BPD reflects a fast “Pace-of-Life-Syndrome” (PoLS). Ninety-five women (44 diagnosed with BPD) were recruited to examine psychological correlates of PoLS, including life history features, personality dimensions, aggressiveness, chronic stress, borderline symptom severity, childhood trauma, and allostatic load (AL). In line with expectations, BPD patients had significantly higher scores suggestive of a fast PoLS than controls, they were more aggressive, more burdened with chronic stress and were exposed to more severe childhood adversity. Childhood trauma predicted PoLS, which in turn predicted AL. The present study thus provides direct evidence of psychological and somatic traits associated with the fast end of the PoLS spectrum in females with BPD. Findings are discussed with regard to clinical implications.

## Introduction

Borderline Personality Disorder (BPD) is a psychiatric disorder featured by intense fears of abandonment, difficulties in emotion regulation, feelings of emptiness, unstable interpersonal relationships, impulsivity, and heightened risk-taking behaviors, as well as high levels of interpersonal aggression. In addition, paranoid ideation and dissociative states may occur. Many patients with BPD also show recurring self-injurious or suicidal behavior, although “self-cutting” is not specific to BPD (American Psychiatric Association, 2013). BPD is the most prevalent personality disorder with a reported lifetime prevalence of about 1.7 percent (Gunderson et al., 2018). In clinical settings, BPD is even more frequent, with an 8.3-fold higher all-cause mortality compared to the general population (Kjær et al., 2018), rendering the condition as highly relevant for public health. Although controversially discussed, it seems that females are more frequently affected from BPD, at least in clinical settings, than males, with a ratio of about four to one (Paris et al., 2013).

Etiological models of BPD suggest that the development of “mistrustful inner working models” in relation to insecure attachment patterns predisposes to perceiving others as untrustworthy and rejecting (Fonagy et al., 2000; Agrawal et al., 2004). Causal factors in this development include emotional neglect and physical or sexual abuse, which occur in up to 80 percent of individuals with BPD (e.g., Zanarini et al., 1989; Hurlbert et al., 1992; Bandelow et al., 2005). Indeed, according to Linehan’s theory, a developmental pathway leading to BPD begins with early vulnerability, initially expressed as impulsivity, based on which increased emotional sensitivity emerges (Crowell et al., 2009). As regards genetics, findings are more inconclusive, even though some studies show high heritability of BPD features (Amad et al., 2014). According to Linehan’s theory, BPD arises from a complex interplay between heritable biological vulnerability and an invalidating social environment (Linehan, 1993; Crowell et al., 2009).

Consistent with Linehan’s (1993) and attachment-related theories of BPD, evolutionarily grounded explanations have posited that some signs and symptoms associated with BPD can be seen as a pathological variant of adaptive responses to early environmental adversity (Chisholm, 1999; Chisholm et al., 2005; Brüne et al., 2010; Brüne, 2016). This view is, in part, based on Life History Theory (Stearns, 1992), suggesting that individual differences in the allocation of resources to somatic growth or reproduction depend on an unconscious evaluation of future resource availability (Ellis et al., 2009). Accordingly, if resources (which here includes emotional availability and trustworthiness of significant others) are predicted to be scarce, individuals may tend to adopt a “faster” life history strategy (LHS), i.e., investing more in reproductive activity than body maintenance and tissue repair (Ellis et al., 2009, 2011; Griskevicius et al., 2011), whereby early social stress in the form of abuse or neglect may “prepare” the individual at the neurobiological level to deal with future threat and malevolence (Teicher et al., 2016; Troisi, 2020). The distinction between “fast” and “slow” LHS is by no means dichotomous; the terms rather reflect the extremes on a continuum, with changes over the lifespan that critically depend on environmental cues (Stearns and Rodrigues, 2020).

On a psychological level, a “fast” LHS or “Pace-of-Life-Syndrome” (PoLS), which we here use interchangeably (Dammhahn et al., 2018) would also entail greater impulsivity, higher scores in neuroticism, lower empathy (or agreeableness), higher aggressiveness, less investment in own offspring, yet a tendency toward risky sexual behavior, and frequent disruptions of intimate relationships (Del Giudice, 2016) all of which are prominent characteristics of BPD (Brüne, 2016). This compilation of psychological factors reflecting a “fast” PoLS is also compatible with empirical findings proposing a so-called “Super-K” factor subsuming the “Big Five” personality traits, positive affect, social support, aggression, education, pair-bonding, and physical and mental health. The term “Super-K” refers to the biological concept behind Life History Theory and concerns resource allocation to the timing of reproduction and somatic growth. It thus alludes to the continuum between slow and fast LHS (Richardson et al., 2017).

As regards BPD, several lines of research have corroborated the interpretation that BPD as a clinical syndrome largely echoes a fast PoLS, including studies into sociosexuality and mating (Alvergne et al., 2010; Brüne et al., 2017; Sansone et al., 2011), teenage pregnancy, and number of offspring (De Genna et al., 2012). Moreover, personality traits as well as temperament of people with BPD are compatible with the idea of a fast PoLS (Fossati et al., 2001; Láng, 2015; Del Giudice, 2012, 2016; Dammhahn et al., 2018).

Aside from psychological signs and symptoms associated with BPD, there is evidence to suggest that individuals with BPD are also at heightened risk of developing cardiovascular disease and stroke; in fact, the risk for cardiovascular disease and stroke seems to be even specific for BPD in comparison with other personality disorders (Moran et al., 2007), and largely independent of comorbid depression (Barber et al., 2020). The risk for cardiovascular disease, in part, relates to elevated rates of overweight and obesity, as well as hypertension and diabetes mellitus among people diagnosed with BPD (Powers and Oltmanns, 2012). However, it may also reside in the fact that BPD patients prematurely accumulate a higher “allostatic load” (AL) due to a dysregulated stress response (McEwen, 2000; Boyce and Ellis, 2005). Moreover, there is clear evidence to suggest that childhood adversity poses a risk for developing metabolic syndromes (Lee et al., 2014) and can exert life-long effects on stress responsivity (Boyce, 2014). Together, a larger AL could be a somatic consequence of a “fast” PoLS due to reduced investment in body maintenance and repair.

The concept of “allostatic load” entails that an over-burdened stress-coping system, foremost the hypothalamic-pituitary-adrenal axis, causes the body to accrue negative consequences of maladaptive stress responses in the form of somatic disease (McEwen, 2000). Typical somatic marker for allostatic load comprises morphological parameters, such as the waist-to-hip ratio and body mass index (BMI), and blood serum markers, such as C-reactive protein, the ratio of low-density to high density lipoprotein (LDL/HDL ratio), and glycated hemoglobin (HbA_1_c), as well as systolic and diastolic blood pressure (DBP; Seeman et al., 2001).

In line with Life History Theory, elevated markers for allostatic load in people pursuing a “fast” PoLS can be predicted on the basis of the “disposable soma” hypothesis (Kirkwood and Rose, 1991). The disposable soma hypothesis is similar to the “antagonistic pleiotropy” hypothesis, suggesting that investment in reproduction early in life may be related to earlier deterioration of body functions and senescence due to lower investment in maintenance and repair of tissue damage (Hammers et al., 2013).

### Hypotheses

In summary, current evidence seems to suggest that many features associated with BPD are consistent with characteristics of a fast PoLS (Brüne, 2016; Dammhahn et al., 2018). However, to the best of our knowledge, no study has directly examined LHS in BPD using specific scales designed for this purpose (Figueredo, 2007), measures of physical and mental health in relation to childhood adversity and stress exposure. From a clinical and public health perspective, such insights could be highly relevant in relation to early detection of risk factors for the development of emotionally instability and prevention of poor outcome of BPD.

Accordingly, we sought to explore the PoLS of a female cohort of BPD, how this relates to the exposure to early adversity, current stress perception, and physical health. Specifically, we (1) hypothesized that individuals with BPD would typically display features of a fast PoLS, relative to a control group not affected from the disorder (2) We also expected that a faster PoLS would be statistically predicted by childhood adversity as a causal factor driving this development, and (3) that the clinical group would display a higher allostatic load as an indicator of poor body maintenance and tissue repair.

## Materials and Methods

The study was approved by the Ethics Committee of the Medical Faculty of the Ruhr University of Bochum, Germany, according to The Code of Ethics of the World Medical Association (Declaration of Helsinki) for experiments involving humans. All participants gave fully informed consent in writing.

### Participants

Ninety-five women (mean age 25.9 ± 4.6 years) participated in the study. Among these, forty-four female patients (mean age 26.4 ± 5.5 years) were recruited from an in-patient unit of the LWL University Hospital Bochum, Ruhr University Bochum, Germany with a treatment focus on dialectical behavior therapy. All patients were diagnosed with BPD according to DSM criteria and a structured diagnostic interview (German version by Wittchen and Fydrich, 1997). In addition, a group of 51 women (mean age 25.4 ± 3.7 years) not affected by any psychiatric condition as ruled out by a standardized diagnostic interview, the Mini-Diagnostisches Interview für Psychische Störungen (Mini-DIPS; Margraf, 1994), took part. They were recruited *via* advertisement.

### Assessment

#### Arizona Life History Battery

The Arizona Life History Battery (ALHB) is composed of different original sources that measure subjects’ cognitive and behavioral characteristics suggestive of a fast versus slow LHS (Figueredo, 2007). The component scales comprise “Insight, Planning, and Control,” “Mother/Father Relationship Quality,” “Family and Friends Social Contact,” “Family and Friends Support,” “Experiences in Close Relationships,” “Altruism,” and “Religiosity.” Most of these questionnaires use a 7-item Likert scale to determine how much the participant agrees, respectively, disagrees, with the statement given in each item. Since the majority of participants had no children (38 patients and 44 controls), we excluded the subscale tapping into “altruism toward own children.” In addition, due to cultural differences in religiosity between Germany and the United States, this ALHB subscale was also discarded from further analyses. A higher ALHB score is indicative of a slower LHS. Cronbach’s alpha for the subscales used in the present study is reported to lie between 0.90 and 0.94 for an American college sample (Olderbak et al., 2014).

#### Neuroticism-Extraversion-Openness Five Factor Inventory

The Neuroticism-Extraversion-Openness Five Factor Inventory (NEO-FFI; Costa and McCrae, 1992) is a self-report measure of the “Big Five” personality traits comprising “neuroticism,” “extraversion,” “openness to experience,” “agreeableness,” and “conscientiousness.” The NEO-FFI consists of 60 items that are grouped into five subscales, one for each trait. All subscales have acceptable to good internal consistency (*α* ranges from 0.75 to 0.83).

#### Buss–Perry Aggression Questionnaire

A German version of the Buss–Perry Aggression Questionnaire (BPAQ) was used to measure four different dimensions of aggression: “physical” and “verbal” aggressiveness; “anger” and “hostility,” whereby scores of individual items were summed-up (Werner and von Collani, 2004).

#### Childhood Trauma Questionnaire

The Childhood Trauma Questionnaire (CTQ) consists of 28 questions that are categorized into five subdomains. These are “emotional abuse,” “physical abuse,” “sexual abuse,” “emotional neglect,” and “physical neglect.” The CTQ also considers the tendency of the subject to deny or downplay the experience of childhood abuse. We applied a German version of the CTQ with satisfactory psychometric properties (Cronbach’s alpha 0.8), with the exception of the “physical neglect” scale (Cronbach’s alpha 0.4; Klinitzke et al., 2012). For the purpose of this study, a CTQ sums score was calculated from all items except those concerning denial, as these values were overall low in both the clinical group as well as in controls.

#### Trier Inventory of Chronic Stress

The Trier Inventory of Chronic Stress (TICS) measures how often a certain situation or experience has occurred to the participant in the last three months. The TICS comprises nine subscales, i.e., “work overload,” “social overload,” “pressure to succeed,” “dissatisfaction with work,” “excessive demands at work,” “lack of social recognition,” “social tension,” “social isolation,” and “chronic concern” (Schulz et al., 2004). Cronbach’s alpha of the subscales is between 0.76 and 0.91. For further analyses, we used the TICS sum score.

#### Borderline Symptoms List-23

The Borderline Symptom List (BSL; Bohus et al., 2009) measures symptoms severity. We used the short 23-item version (derived from the 95-item long version) examining “self-perception,” “affect regulation,” “auto-aggressive behavior,” “dysphoria,” “social isolation,” “intrusions,” and “hostility” on a 5-point Likert scale, as well as the current emotional situation. The BSL-23 has excellent reliability (Cronbach’s alpha = 0.935–0.969; Bohus et al., 2009).

#### Allostatic Load

Anthropological measures available for the entire sample included waist and hip circumference, systolic and diastolic blood pressure (SBP and DBP), weight and height, from which waist-to-hip ratio, and BMI were calculated. SBP and DBP were measured with seated blood pressure reading. For the waist circumference, the narrowest point between the ribs and the iliac crest was chosen. The hip circumference was measured over the buttocks. Allostatic load (AL) was determined as a composite score comprising systolic blood pressure (SBP ≥ 148 mm Hg), diastolic blood pressure (DBP ≥ 83 mm Hg), waist-to-hip ratio (≥ 0.85), and BMI (≥ 23.5). The cutoff values were adopted from Seeman et al. (2001) and Juster et al. (2010), respectively. In addition, we explored an alternative approach as suggested by Seeman et al. (2001) where sample-specific cutoff points were determined by classifying subjects’ anthropometric measures (i.e., SBP, DBP, BMI, and WtH) into quartiles. AL was measured by summing the number of parameters for which the subject fell into the top quartile (thus, the AL score ranged from 0 to 4 points).

### Statistical Analyses

All analyses were carried out using the commercial statistics software IBM SPSS Statistics, Version 26 (IBM Corp., Armonk, NY, United States). Statistical significance levels were set at *p* < 0.05, adjusted for multiple comparisons where appropriate. Group comparisons were calculated using multivariate analyses of variance (MANOVA). For correlational analyses, we report Pearson’s coefficient values. To further study the relationship between childhood trauma, PoLS and AL, we performed a mediation analysis using the macro tool PROCESS for SPSS developed by Hayes (2018). Accordingly, the CTQ score (X), PoLS (M), and AL (Y) were fed into the equation, whereby indirect effects were estimated by bootstrap analysis with 5,000 samples.

## Results

### Comparisons Between Groups

The group diagnosed with BPD and the control group were carefully matched for age (and sex), such that no statistically significant difference emerged with regard to age (*F* = 0.951, df = 1, *p* = 0.332).

As expected, a MANOVA revealed that the two groups differed significantly in all aspects of LHS as measured using the ALHB (Table 1). That is, the corrected model was also highly significant for the ALHB sum score (*F* = 209.456, df = 1, *p* < 0.001). Similarly, highly significant group differences occurred for symptom severity as measured using the BSL-23, the “Big Five” personality traits (NEO-FFI), with the difference in “openness” being less significant than the other four factors, aggressiveness (BPAQ), and chronic stress exposure (TICS), for which all values of *p* were below 0.001, after correction for multiple comparisons (shown in Table 2).

**Table 1:** Comparison of biological and life history data (incl. SD) between the BPD patient group and unaffected controls.

	BPD	Control group	Value of *p*
	(*n* = 44)	(*n* = 51)	
Age (years)	26.4 (5.5)	25.4 (3.7)	n.s.
SBP	119.1 (12.8)	118.8 (12.2)	n.s.
DBP	80.6 (7.3)	73.3 (8.2)	*p* < 0.001
BMI	27.9 (9.0)	21.6 (2.6)	*p* < 0.001
WtH ratio	0.81 (0.087)	0.79 (0.082)	n.s.
AL	1.27 (1.12)	0.5 (0.71)	*p* < 0.001
ALHB insight and planning	−0.063 (1.07)	1.49 (0.54)	*p* < 0.001
ALHB parent relationship	1.47 (0.55)	2.22 (0.38)	*p* < 0.001
ALHB family contact/support	1.57 (0.65)	2.47 (0.39)	*p* < 0.001
ALHB friends contact/support	1.37 (0.76)	2.13 (0.49)	*p* < 0.001
Partner attachment	−0.46 (0.76)	1.48 (0.58)	*p* < 0.001
Altruism	−0.006 (0.77)	0.82 (0.75)	*p* < 0.001
ALHB composite score	0.65 (0.45)	1.77 (0.33)	*p* < 0.001

*SBP, Systolic blood pressure; DBP, Diastolic blood pressure; BMI, Body Mass Index; WtH ratio, Waist-to-hip ratio; AL, Allostatic load; and ALHB, Arizona Life History Battery*.

**Table 2:** Comparison of borderline symptomatology, personality dimensions, childhood trauma, chronic stress, and aggressiveness between patients with BPD and controls.

	BPD	Control Group	Value of *p*
	*N* = 44	*N* = 51	
BSL-23	47.3 (20.3)	4.4 (4.4)	*p* < 0.001
Neuroticism	37.2 (6.3)	16.8 (5.7)	*p* < 0.001
Extraversion	22.9 (7.5)	30.7 (6.0)	*p* < 0.001
Openness	29.7 (6.5)	32.7 (5.7)	*p* = 0.024^*^
Agreeableness	26.9 (5.9)	37.2 (4.9)	*p* < 0.001
Conscientiousness	24.8 (8.1)	34.0 (4.8)	*p* < 0.001
CTQ	58.6 (19.8)	24.5 (4.2)	*p* < 0.001
BPAQ	74.3 (13.2)	42.9 (7.1)	*p* < 0.001
TICS	144.3 (33.0)	70.0 (21.9)	*p* < 0.001

*BSL-23, Borderline Symptom List; CTQ, Childhood Trauma Questionnaire; BPAQ, Buss–Perry Aggressiveness Questionnaire; and TICS, Trier Inventory of Chronic Stress*.

* *not surviving correction for multiple comparisons*.

In addition, we compared AL measures between the groups and found that SBP and WtH ratio did not differ between people with BPD and unaffected controls (both *p* > 0.05). However, the groups differed significantly with regard to the AL composite score (*F* = 15.335, df = 1, *p* < 0.001, corrected for multiple comparisons), independent of whether the cutoff values were used, or specific cutoff points for this sample. As results were almost identical for the two procedures determining AL, we only report findings using the cutoff values (Seeman et al., 2001; Juster et al., 2010).

### Correlations

For correlational analyses, we pooled the data of both groups, as our overall interest was to determine if PoLS would correlate with those factors subsumed under the “Super-K” factor (Richardson et al., 2017), i.e., the “Big Five” personality factors and aggression, chronic stress, childhood trauma, and AL. In line with predictions, we found highly significant correlations between PoLS, the “Big Five” personality traits, symptom severity (BSL-23), aggressiveness (BPAQ), chronic stress (TICS), childhood trauma (CTQ), and AL. Note that lower ALHB scores reflect faster PoLS, which explains why several measures were inversely correlated (shown in Table 3). All of the above-mentioned values remained highly significant after familywise correction for multiple correlations according to Bonferroni-Holm. In addition, AL correlated with age, neuroticism, agreeableness, aggression, and chronic stress. As shown in Table 3, there were multiple correlations (again, corrected for multiple comparisons) between personality traits and scores of childhood trauma, aggressiveness, and chronic stress, as well as borderline symptom severity, all of which were in the expected direction. Note that we also checked non-parametric correlation coefficients for differences to Pearson’s, which were overall negligible or absent. In addition, we performed correlation analyses for AL in two ways: one using the cutoff values as described in Seeman et al. (2001) and Juster et al. (2010), and the other using the sample-specific top-quartiles. The correlation coefficients differed marginally (in the second or third decimal place), such that Table 3 displays only the figures for the cutoff points reported in Seeman et al. (2001) and Juster et al. (2010).

**Table 3:** Pearson correlations of life history strategy with age, allostatic load, borderline symptom severity, childhood trauma, aggressiveness, and chronic stress (data of patients and controls pooled).

	ALHB	Age	AL	BSL-23	Neurot	Extr	Open	Agree	Consc	CTQ	BPAQ	TICS
ALHB												
Age	−0.106											
AL	−0.285^*^	0.352^*^										
BSL-23	−0.795^*^	0.059	0.264									
Neurot	−0.809^*^	−0.006	0.322^*^	0.877^*^								
Extr	0.642^*^	−0.022	−0.085	−0.579^*^	−0.677^*^							
Open	0.384^*^	−0.430^*^	−0.269	−0.189	−0.148	0.228						
Agree	0.639^*^	−0.142	−0.363^*^	−0.611^*^	−0.691^*^	0.437^*^	0.294^*^					
Consc	0.546^*^	0.105	−0.200	−0.516^*^	−0.656^*^	0.394^*^	0.158	0.607^*^				
CTQ	−0.765^*^	0.104	0.170	0.665^*^	0.664^*^	−0.442^*^	−0.167	−0.464^*^	−0.331^*^			
BPAQ	−0.744^*^	0.132	0.412^*^	0.737^*^	0.798^*^	−0.471^*^	−0.308^*^	−0.723^*^	−0.642^*^	0.607^*^		
TICS	−0.677^*^	−0.010	0.346^*^	0.748^*^	0.800^*^	−0.382^*^	−0.146	−0.723^*^	−0.654^*^	0.578^*^	0.775^*^	

*ALHB, Arizona Life History Battery; AL, Allostatic load; BSL-23, Borderline Symptom List; Neurot, Neuroticism; Extr, Extraversion; Open, Openness; Agree, Agreeableness; Consc, Conscientiousness; CTQ, Childhood Trauma Questionnaire; BPAQ, Buss–Perry Aggression Questionnaire; Aggr, Aggressiveness; and TICS, Trier Inventory of Chronic Stress*.

* *Correlation is significant, familywise corrected for multiple comparisons according to Bonferroni-Holm*.

### Mediation Analyses

**Figure 1:**
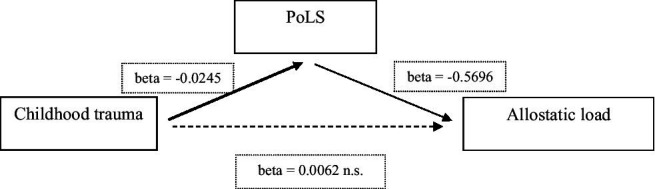
Summary and overview of a mediation analysis to predict PoLS and AL (thickness of arrows represent the magnitude of the statistical effect). PoLS, Pace-of-Life-Syndrome.

To examine direct and indirect effects of childhood trauma and PoLS on AL, we carried out a mediation analysis as described above (data for both groups pooled; Hayes, 2018). The overall model was significant [*F*(2, 88) = 10.35, *p* = 0.017, *R*^2^ = 0.088]. Results indicated that childhood trauma (X) was a significant predictor for PoLS (M) [*b* = −0.0245, *t*(89) = −11.856, *p* < 0.001]. In addition, PoLS predicted AL (Y) [*b* = − 0.5696, *t*(88) = −2.396, *p* = 0.0187]. Furthermore, a bootstrapped analysis revealed an indirect effect of early adversity (X) on AL (Y) *via* PoLS (M) (*a* × *b* = 0.0139 with 95% confidence interval excluding 0 from 0.0028 to 0.0272). However, there was no direct effect of CTQ [*b* = −0.0062, *t*(88) = −0.8356, *p* = 0.406] (Figure 1).

## Discussion

This study set out to examine the evolutionary hypothesis that BPD, the most severe and most frequent personality disorder in the general population, was linked to cognitive, emotional, and behavioral features typical for a fast LHS or Pace-of-Life-Syndrome (PoLS). To this end, we employed several psychological measures tapping into individual differences in PoLS. In essence, this included the examination of psychological LH features, personality traits, aggressiveness, as well as the exploration of physical and mental signs and symptoms that in previous research have loaded on a “Super-K” factor (Richardson et al., 2017). Moreover, we also assessed the experience of childhood trauma as a potential causal factor of a fast PoLS.

In line with previous research indicating the association of a fast PoLS with BPD (Brüne et al., 2010, 2017; Brüne, 2016; Del Giudice, 2016), the direct comparison between BPD patients and controls corroborated these findings. In fact, BPD patients differed significantly from unaffected controls in virtually all facets. Aside from differences in LH features, BPD patients also displayed a personality profile and higher aggressiveness consistent with the “Super-K” model. There was also a significant difference in AL between BPD patients and controls, although not as strong as expected, presumably owed to the young age of the participants.

We also sought to determine more causal relationships among these factors in their statistical weight to predict differences in PoLS and AL. Indeed, Belsky et al. (1991) and others have argued how early adversity may cause a “chain reaction” impacting on the speed of psycho-social development (Belsky, 2019). Along similar lines, Chisholm (1993) has outlined from another angle the relevance of a slow LHS for a healthy emotional and social development, i.e., fostering a slow PoLS (see also Chisholm, 1999). Accordingly, we performed a mediation analysis (Hayes, 2018), whereby childhood adversity was hypothesized to statistically predict PoLS, which in turn was explored with regard to its potential predictive value for the accumulation of somatic consequences of poor stress regulation as proposed elsewhere (Teicher et al., 2016; Troisi, 2020). In fact, we found a significant impact of childhood trauma on PoLS, and of PoLS on AL, but no direct effect of childhood adversity of AL. This finding is consistent with the interpretation of a mediating effect of PoLS on the indirect relationship between early adversity and AL. Consistent with our findings, Barber et al. (2020) have recently reported that people with BPD who are in their mid-40s have an elevated cardiometabolic risk mediated by insulin resistance, adiposity, dyslipidemia, and blood pressure, which seems to clearly reflect elevated AL in this clinical group. In our study, the AL score comprised only four measures (SBP, DBP, BMI, and WtH ratio), while it lacked additional serum markers examining chronic inflammation, dyslipidemia, or insulin resistance. However, as shown in Table 3, AL correlated quite strongly with aggressiveness and chronic stress, as well as to a minor degree with neuroticism and (inversely) with agreeableness, suggesting that body maintenance and repair clearly deserve attention to prevent somatic consequences of PoLS and stress in clinical population (Boyce and Ellis, 2005; McEwen, 2017).

In line with the “Super-K” model, the other factors studied (i.e., “Big Five” personality traits, aggressiveness, and borderline symptom severity correlated with PoLS in the expected direction). For example, Ellis et al. (2012) have argued that aggression can be advantageous for reproductive effort as part of a “fast” LHS. Along similar lines, Hurst and Kavanagh (2016) found in a non-clinical sample that people reporting a “faster” PoLS scored higher on measures of aggression and hostility.

As regards the experience of chronic stress, Table 3 illustrates the high correlations not just with PoLS scores, but also with neuroticism, borderline symptom severity, aggressiveness, and (inversely) with agreeableness. As Troisi (2020) has pointed out, chronic stress can be both, a causal factor and a consequence of a “fast” PoLS. Arguably, a “fast” PoLS is likely linked to a heightened exposure to stressful events. Conversely, experiencing chronic stress may support one’s view that the world is dangerous and untrustworthy, such that more opportunistic attitudes may be the best strategy (no conscious decision making involved).

While the focus of the present research was to explore evolutionarily guided hypotheses derived from Life History Theory, it is important to note that our findings are entirely consistent with other theoretical frameworks, such as the one proposed by Linehan (1993) or attachment-based theories of BPD (Agrawal et al., 2004). Indeed, Life History Theory is considered an “intermediate-level” evolutionary theory that has the potential to guide empirical work on gene-environment interaction, including associations between heritable traits and maladaptive environmental conditions (Troisi, 2020). While the present study did not provide information about heritability or genetic factors involved in the association of an individual’s responsivity to early adversity and its interaction with maladaptive coping, we cannot infer causality, even though our findings seem to support the view that childhood trauma can have pervasive effects on one’s psychosocial behavior and somatic consequences in the long run (Boyce, 2014).

The present research has several shortcomings. First, we used a composite score of features tapping into psychological correlates of individual differences in LHS (i.e., the ALHB). The ALHB has, however, been criticized for its conflation of proximate and ultimate factors (Gruijters and Fleuren, 2018), while others even dispute that humans display coherent LHS or PoLS (Sheppard and Van Winkle, 2020). Moreover, there is an obvious disparity how the concept of Life History is used in evolutionary biology (focusing on species-typical patterns) versus evolutionary psychology (emphasizing individual differences; Nettle and Frankenhuis, 2020; Stearns and Rodrigues, 2020) While this debate cannot be settled here (for a defense of the approach, see Figueredo et al., 2015) one obvious shortcoming of the present study is the lack of biological measures suggestive of a fast LHS, including ones concerning somatic growth, mating, reproduction, and lifespan. However, we have previously reported that mating and sexual behavior of females with BPD are also compatible with a Life History Theory approach (Brüne et al., 2017). Moreover, the present research was cross-sectional by design, and therefore not ideally suited to study developmental issues, where a longitudinal design would be optimal. Along similar lines, retrospective rating of childhood adversity can be fraught with the problem of overstating neglect or abuse, particularly in individuals who, at the time of assessment, face psychological problems (Colman et al., 2016; Nivison et al., 2021). Another shortcoming of the present work is the lack of control for socioeconomic status. Indeed, one could argue that individuals with BPD are, on average, socioeconomically disadvantaged, like many other people with mental illnesses, compared to a psychologically healthy control group. However, the question of chicken and egg is open, and the argument can become circular. Low socioeconomic status increases the risk for early adversity (Belsky et al., 1991), and hence the risk for assuming a faster LHS. Conversely, in the case of BPD, difficulties in coping with the disorder may perpetuate the poor socioeconomic situation in a vicious circle manner. In any event, without going into further detail here, future studies should consider socioeconomic status (Muscatell et al., 2020). A third point concerns sex differences. In the present study, only female subjects were recruited. It is known that males and females respond differently to stress, with females being at greater risk to early life and peripubertal adversity (Bale and Epperson, 2015; Troisi, 2020), such that the present findings are not generalizable. Another shortcoming concerns the way AL was measured. As stated above, in the present study AL only comprised blood pressure, BMI, and WtH ratio, while other important metabolic and inflammatory measures were not available, which would be desirable to obtain in future studies. Finally, ideally the study would have included a clinical comparison group suggestive to pursue a “slow” PoLS (Del Giudice, 2016). Likewise, we believe that a fast PoLS is by no means specific to BPD; instead, there are several psychiatric conditions, including attention deficit/hyperactivity disorder, bipolar disorder, or addictive disorders that fall into this category. Of note, comorbidity among these with BPD is particularly high (Shah and Zanarini, 2018).

## Conclusion

In summary, to the best of our knowledge, this is the first study that has directly examined the question of whether the clinical condition labeled “Borderline Personality Disorder” bares features suggestive of a fast PoLS and consequences including poorer body maintenance and repair. The findings could therefore be of particular relevance for public health, particularly in terms of prevention and risk reduction for deleterious outcomes that are not only psychologically determined, but also by means of physical health. Moreover, this research demonstrates, in a more general vein, the usefulness and applicability of evolutionary thinking in clinical medicine.

## Data Availability Statement

The raw data supporting the conclusions of this article will be made available by the authors, without undue reservation.

## Ethics Statement

The studies involving human participants were reviewed and approved by Ethics Committee of the Medical Faculty of the Ruhr University Bochum, Germany. The patients/participants provided their written informed consent to participate in this study.

## Author Contributions

BO and LK: acquisition of the data. BO and MB: analysis, interpretation of the data, and drafting the article. LK and MB: revision of manuscript for important intellectual content and study design. BO, LK, and MB: final approval of the version to be submitted. All authors have approved the final article.

## Conflict of Interest

The authors declare that the research was conducted in the absence of any commercial or financial relationships that could be construed as a potential conflict of interest.

## Publisher’s Note

All claims expressed in this article are solely those of the authors and do not necessarily represent those of their affiliated organizations, or those of the publisher, the editors and the reviewers. Any product that may be evaluated in this article, or claim that may be made by its manufacturer, is not guaranteed or endorsed by the publisher.
